# Identifying Stops and Moves in WiFi Tracking Data

**DOI:** 10.3390/s18114039

**Published:** 2018-11-19

**Authors:** Cristian Chilipirea, Mitra Baratchi, Ciprian Dobre, Maarten van Steen

**Affiliations:** 1 University Politehnica of Bucharest, Romania, Computer Science Department, Splaiul Independenței 313, 060042 Bucharest, Romania; ciprian.dobre@cs.pub.ro; 2 Leiden University, Rapenburg 70, 2311 Leiden, The Netherlands; m.baratchi@liacs.leidenuniv.nl; 3 University of Twente, 7522 Enschede, The Netherlands; 4 ICI Bucharest, Bulevardul Mareșal Alexandru Averescu, 011454 Bucharest, Romania

**Keywords:** crowd movement analysis, trajectory data mining, WiFi tracking, mobility modeling

## Abstract

There are multiple methods for tracking individuals, but the classical ones such as using GPS or video surveillance systems do not scale or have large costs. The need for large-scale tracking, for thousands or even millions of individuals, over large areas such as cities, requires the use of alternative techniques. WiFi tracking is a scalable solution that has gained attention recently. This method permits unobtrusive tracking of large crowds, at a reduced cost. However, extracting knowledge from the data gathered through WiFi tracking is not simple, due to the low positional accuracy and the dependence on signals generated by the tracked device, which are irregular and sparse. To facilitate further data analysis, we can partition individual trajectories into periods of stops and moves. This abstraction level is fundamental, and it opens the way for answering complex questions about visited locations or even social behavior. Determining stops and movements has been previously addressed for tracking data gathered using GPS. GPS trajectories have higher positional accuracy at a fixed, higher frequency as compared to trajectories obtained through WiFi. However, even with the increase in accuracy, the problem, of separating traces in periods of stops and movements, remains similar to the one we encountered for WiFi tracking. In this paper, we study three algorithms for determining stops and movements for GPS-based datasets and explore their applicability to WiFi-based data. We propose possible improvements to the best-performing algorithm considering the specifics of WiFi tracking data.

## 1. Introduction

Making sense of crowd tracking data is far from trivial. Individuals can have unique movement behaviors, while some crowd-level characteristics can be maintained. It is even more difficult to make sense of this type of data when positions are approximate and detections are sparse.

To simplify tracking data, we can separate them into periods of stops and moves [1,2]. This is a fundamental step that can be used to answer many questions that would be intangible given the raw dataset. A few possible questions are: “What are the most interesting locations?”, “How many people are traveling in pairs or small groups?”

In this paper, we explore the problem of splitting an individual’s trace into periods of stops and movements. We concentrate on WiFi tracking, a specific implementation of mobility data collection using radio frequency signals, which takes advantage of the fact that WiFi devices are ubiquitous and always with us. We expect that the discussed methods can easily be implemented for other radio frequency technologies, such as Bluetooth.

The problem of distinguishing stops from movements is not new, as it has already been explored for GPS-tracking. When visualizing GPS datasets, stop periods appear as positions randomly placed around the stop location. Because of this, some algorithms that detect stops are based on clustering methods. We have identified three such methods in the existing literature: stay point detection [3,4], Cbsmot [2] and Dbsmot [5]. These methods utilize different properties of a trace: direction, speed, and distance. Unlike GPS traces, WiFi traces are sparse and have a positional accuracy of about 100 m. GPS positioning reaches decimeter accuracy, and devices can record positions with a high frequency. These differences can translate into different performance of the algorithms on WiFi datasets.

For large-scale crowd monitoring, technologies based on the detection of smartphones prevail. The most popular ones make use of call records [6] and WiFi sensing [7]. Using call records scales better, but the data are sparser because detections are recorded only when a person makes a phone call or sends an SMS. The positioning accuracy is in the order of kilometers, the range of the GSM tower. In contrast, WiFi has a limited communication range, of about 100 m. WiFi-enabled smartphones also transmit more, as they try to connect to different networks or as installed apps try to communicate over the network, increasing their chances of being detected.

WiFi tracking is performed by using a set of sensors deployed across the detection area. The sensors are simple WiFi-enabled devices, such as WiFi routers, specifically configured to record detections of WiFi devices (smartphones). The sensors listen for all WiFi frames and extract the MAC address from them. The time, hashed MAC address (for privacy reasons), and the position of the sensor form a tuple that describes a detection. By having a set of detections for one device, we can trace its path through the area covered by the sensors.

When using WiFi tracking, the tracked objects do not participate actively. WiFi devices are configured by default to send WiFi frames regularly, exposing the device address and location. The unobtrusiveness of the method and the pervasiveness of smartphones make WiFi tracking scalable and inexpensive to deploy. Unfortunately, the sparsity and low accuracy of WiFi tracking datasets get even worse when we are dealing with large crowds. The human body blocks the electromagnetic signal, and signals from multiple devices interfere with each other. WiFi devices make use of low-level collision avoidance techniques to communicate even under high interference. However, sensors are not part of the normal WiFi communication and cannot make use of these techniques.

In the next section, we go into detail on what the limits of WiFi tracking are and how the sparsity and low accuracy positions affect the analysis. Furthermore, we will better describe what some of causes of the sparsity of data are.

To our knowledge, we are the first to measure the accuracy of stop/move partition algorithms on datasets constructed using RF-signals, particularly WiFi. Previous work has analyzed WiFi traces [8], but for specific contexts, for datasets gathered indoors, where they could make use of the received signal strength indicator (RSSI) to perform trilateration and improve the accuracy of positioning. Performing WiFi tracking in outdoor settings, we have realized that in complex situations (such as the presence of large crowds), RSSI values are too erratic and devices are rarely simultaneously detected by enough sensors to be able to apply trilateration. Furthermore, we prove that for the types of datasets that we explore, it could be impossible to achieve perfect accuracy in determining stops and moves. Even with knowledge of the correct labeling, the sparsity of the data does not permit partitioning the trace so that each second is labeled correctly.

Lastly, we bring improvements to the most promising of the three methods, stay point detection. These improvements make use of the tracking dataset to build a better estimate of the closeness of sensors, as opposed to the Euclidean distance. As an advantage, without the Euclidean distance, the location of sensors is not needed, easing the deployment of tracking platforms, where for example routers acting as sensors are already deployed and their location is not correctly recorded.

## 2. WiFi Tracking Datasets and Their Limitations

A WiFi tracking platform consists of several sensors. The sensors’ locations are known and are used to estimate the position of detected devices. These platforms make use of the assumption that most people carry a WiFi-enabled device such as a smartphone.

Building new WiFi sensing platforms is inexpensive. There are multiple, inexpensive options for devices with WiFi that can act as sensors. Furthermore, WiFi networks with multiple WiFi access points or WiFi routers can easily be configured to act as a WiFi tracking platform.

The sensors are set to listen for all WiFi frames (as defined by the IEEE 802.11 standard) and to record the time, encrypted address and the sensors’ own positions. The tuple <time, address, position> represents a detection, and the set of all detections, from all sensors, for a device represents the device’s trace.

Not all frames sent by devices are captured. For a frame to be captured, the device needs to be close enough to the sensor so that the signal is strong enough when it reaches it. The ideal range for WiFi is 100 m, but it is reduced because of obstructions or it is extended due to tunneling effects. Only frames that are complete and correctly decoded can be recorded; faulty frames are dropped by the network module or the operating system. Interference from other WiFi devices broadcasting at the same time or noise from the environment disrupts the WiFi frames and makes it impossible for them to be correctly captured.

The range at which WiFi signals are detectable is determined by the transmission power and the antenna. There is a high variation between devices based on manufacturer, impurities in the metals that form the antenna, and the software running on the devices. In some cases, the frequency can even be affected by the battery level, installed applications or the screen status of the mobile device. This has been shown in the work by the authors of [9]. In contrast, GPS tracking can be done with a frequency that is constant and high.

Our own experiments and a review of the literature revealed that for indoor scenarios, the frequency with which frames can be correctly detected increases. For 90% of the data acquired from indoor WiFi tracking [1], the detection rate was less than one second. In contrast, for our outdoor experiment, with a high number of people, only 20% of the data had an inter-detection time of less than one second.

We performed a large-scale experiment in the city of Assen, The Netherlands, in 2016. The data were gathered during a festival that attracted more than 150,000 tourists. With such a large crowd, it was expected that WiFi quality would drop. Part of this drop is caused by an abundance of control frames, which use up a large portion of the available bandwidth.

**Figure 1:**
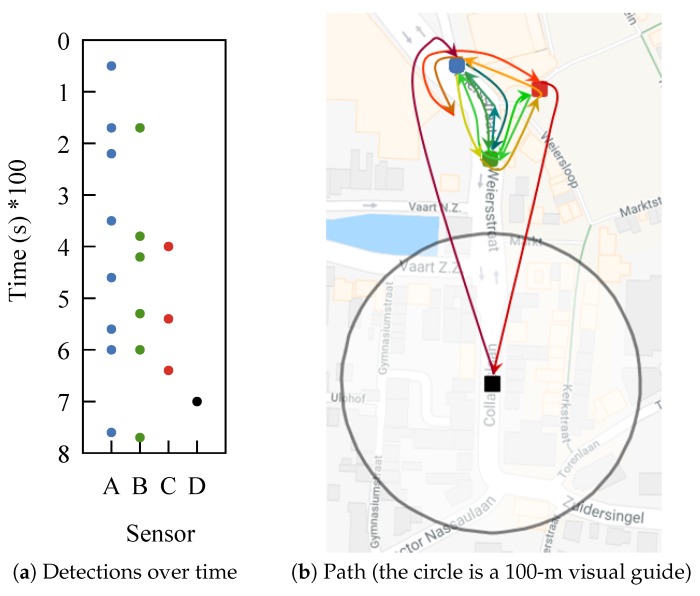
Irregular movements of a static device (artificial trace based on real ones).

One example from our dataset shows how difficult it can be to make sense of WiFi tracking data. Take a person that is positioned between three sensors, sitting there for a long time. The device carried by this person is detected by the three sensors, but almost never at the same time. The time between detections is in the order of minutes, as can be observed in Figure 1a. This makes it impossible to apply trilateration. What is worse is that at some point, the device is detected by a fourth sensor. If we trace the path based solely on detections, we obtain the one from Figure 1b. The device, although most likely static, appears to be moving chaotically. Most of our dataset has devices that exhibit this type of behavior.

A similar behavior to what we observed in Figure 1 was noted in [10], where it was called the “ping pong” effect, symbolizing this back and forth movement.

We have discovered that throughout the dataset, less than 4% of detections represent ones that have triggered at least two sensors simultaneously and less than 0.5% have the same sequence number. This means that 3.5%, although detected simultaneously, represent detections of different frames. This is possible because multiple frames are sent every second, and our system was configured to record the time with a precision of one second.

Applying trilateration on the part of the data with simultaneous detections is not feasible. To apply trilateration, we need an estimate of the distance between each sensor and the device. In theory, the RSSI decreases as the distance between the sensor and tracked device increases. However, in our dataset, RSSI values have a high variation. RSSI has values between −80 and −20, and for devices that we determined to be static (through visual analysis and use of the manufacturer identifier), it has a mean standard deviation of nine. We expected these devices to almost always be detected with the same RSSI. Even worse, transmission power is different for each device, meaning we cannot compare values from different devices.

RSSI quality is dependent on the environment. The authors of [11] managed to use RSSI and trilateration to determine how much time people spend together in a dining hall. Because there were only a few individuals and the experiment took place indoors, the data were less noisy. The authors had 95% of detections at less than two minutes between them, while we have only 83% of detections with a gap smaller than two minutes. Although the difference does not seem significant, gaps add up when considering the sparsity of the data.

We analyzed the source of sparsity from WiFi-tracking datasets with a simple experiment. In one of our offices, we set up two WiFi sensors and configured them to record all the frames they received. The sensors were placed on a table, 50 cm apart.

**Figure 2:**
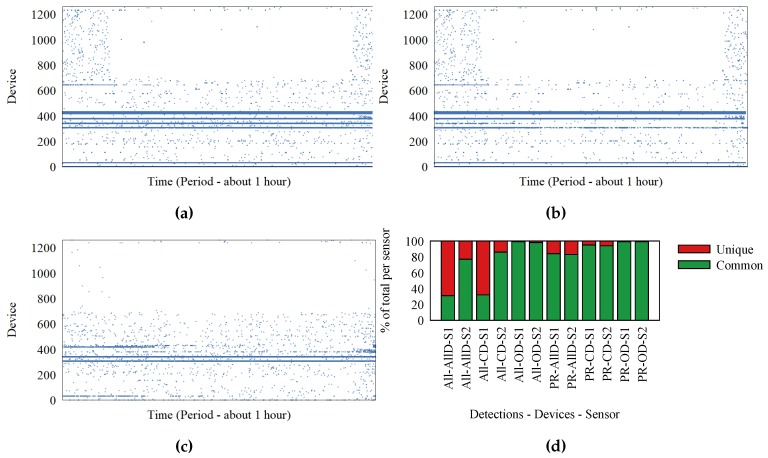
Office experiment: detections at two sensors placed 50 cm apart. (**a**) Sensor 1; (**b**) Sensor 2; (**c**) (S1−S2)∪(S2−S1), S1 and S2 are detections at sensors; (**d**) differences between the sensors. AllD, all devs; CD, common devs; OD, our devs. All, all frame types; PR, only probe requests.

The two sensors recorded frames for one hour, on the same WiFi channel. Figure 2a,b represents the detections at Sensors 1 and 2, respectively. Each dot represents a detection; the Ox axis represents time; and Oy represents the device that transmitted the frame. We identify the devices based on the MAC address inside the frame. It is possible for MAC addresses to be randomly generated, but for simplicity, we assume each MAC address corresponds to a device. These two figures show a significant difference between what the two sensors detect. To better visualize this difference, we extracted the set of detections made at only one of the sensors and represent them in Figure 2c. In ideal circumstances, Figure 2c would be empty. What the graphs do not show is a large number of detections for devices (probably just MAC addresses) observed by only one of the sensors. These detections represent a significant part of the data, and adding them would have cluttered the graphs.

Because the sensors are placed close to each other, we expect them to receive almost the same frames. Frames detected by one sensor, but not the other exist because of interference and environment noise, which cause the signals to be malformed, resulting in frames that cannot be decoded and recorded.

The percentage of lost frames is different from device to device, based on position and environment. Eight devices were inside the same office with the sensors. These were laptops, tablets, and smartphones. We name them “our devices” (OD). Devices detected by both sensors are called “common devices” (CD). Finally, we have the group of “all detections”. We do not know anything about devices outside the initial group of eight inside the office.

In Figure 2d, we show with red what percentage of detections are recorded at only one of the sensors. Each bar represents one sensor and a specific subset of detections. There are two groupings for detections. One contains “our devices”, “common devices”, and “all devices”. The other grouping contains all frames, as opposed to only probe request frames.

Devices outside the office, which have the highest chance of being affected by environmental conditions, are the ones that have the lowest number of detections recorded at both sensors. The percentage of unique detections reaches 70%, meaning most frames are not received by both sensors. The percentage of detections at both sensors is improved when we consider only probe request frames. It is possible for the communication stream to trigger many detections, all detected at only one sensor. Probe request frames are not part of normal communication, as they are control frames.

The conclusion that most frames are not detected is supported by the 50% of frames we observed during our experiment with the retransmission flag set to true. In the outdoor festival environment, frame loss can be even higher. When conducting WiFi tracking, the high frame loss causes the sparsity in traces. Furthermore, sensors that have overlapping detection areas are placed at more than 70 m apart, making it unlikely that frames sent by devices in the overlapping area are detected by both sensors. This creates the appearance of a back and forth movement like the one from the trace in Figure 1b.

## 3. Related Work

Analysis of WiFi traces has been an interesting research topic in the past few years. Initially, the scope was limited to counting the number of people in an area, like [12], which went into detail on validating the counts obtained from WiFi with the real number of people. Counting is a simple tool that can offer answers only to a small set of questions.

One way to answer more complex questions using tracking data is to combine them with domain knowledge. This is the method used in [13], where schedules and known behavior characteristics of personnel were combined with tracking data to determine facility planning. Unfortunately, domain knowledge is not always available, and when it is, it offers only application-tailored solutions.

Information on crowds can be extracted when analyzing very large amounts of tracking data, in the order of months or years. Analysis can be done at low resolution, which hides the sparsity of the dataset. In [14], people are grouped with the use of SSIDs (identifiers of networks they connected to in the past) and low-resolution presence matrices (columns are time, and rows are locations), with a time resolution of one hour. When analyzing a festival that lasts a few days, using such a resolution would not leave enough data from which to extract relevant information.

Using datasets that expand over a long time can offer very interesting results: the authors of [15] showed how relationships can be extracted along with their strength, for both humans and animals; while [16] proposed a way to identify groups by analyzing the time people enter and exit a room. The idea here is that people can share a location (such as a restaurant) without being part of the same group, but the time at which they arrive and leave reveals more about their structure. This idea is in line with our goal of discovering stops and movements, which can be used to answer this type of query efficiently.

The need to offer methods of extracting information from data that offer a large-scale view, over a city center, but for a relatively short time frame, such as that of a festival, exists. For instance, in [17], the authors gathered data during an event very similar to the one we analyze. They set up a tracking platform using Bluetooth, during a festival in Denmark, which, like the one we analyze, had multiple stages for performers. The advantage of using Bluetooth is that it has a smaller communication range, allowing more accurate positioning information. The disadvantage is that most devices have Bluetooth turned off by default, and fewer individuals turn it on compared to those who use WiFi. This is also observed from the size of the datasets. The authors reported the identification of only 10%, as many devices as we detect in a similarly-sized festival. The tracking technologies are very similar, but we would argue that WiFi tracking offers a more complete picture, to the detriment of positional accuracy.

## 4. Determining Periods of Stops and Movements

A trace of an individual extracted from a tracking dataset consists of a set of timestamped locations. When these points have a regular, high frequency and high positional accuracy, placing them on a map reveals the paths taken by the individual, the places and the time at which they were visited. However, when the data are sparse and the positioning accuracy is low, as is the case for WiFi traces, making any sense of the data in this raw form is difficult.

An important step in simplifying traces is to partition the data into stop and move periods. We have selected three algorithms that can partition traces generated using GPS data.

We chose these three algorithms because they use different attributes of the movement data: distance, speed, and direction. By analyzing them on the datasets we obtained using WiFi tracking, we aim not only to identify which solution fits best, but also to understand which attribute is more useful in understanding this type of dataset. The algorithms are:

- Cbsmot [2], a modification of the popular clustering algorithm Dbscan. Cbsmot uses the time and distance between consecutive detections. This means the algorithm can detect areas when the speed is low.
- Dbsmot [5] is inspired by Cbsmot, but instead of taking speed into account, it takes the direction of movement. The idea is that when the device is moving, detections appear in a somewhat straight line, but when it is standing still and detections are formed around it, drawing lines from one detection to the next appears as a device that is greatly changing direction.
- Stay Point detection [3,4] is based on the idea that points generated when a device is static are bounded to an area based on a distance given by the accuracy of the measuring technique. When a device has a new position further than this set limit, it must have moved. We take a pivot in the first point in the dataset and update the pivot when a new, far enough location is found.

## 5. Algorithm Comparison

Our aim is to determine the feasibility of using the three algorithms developed for GPS tracking on tracking datasets obtained using WiFi tracking. To do this, we obtained a WiFi-tracking dataset on which we tested the three algorithms.

### 5.1. Dataset

We built a WiFi-tracking platform by placing 40 sensors in the city of Assen, The Netherlands. The sensors collected WiFi tracking data during the TT Festival (https://www.ttfestival.nl/en/ (27 June 2017)) in 2016. We have previously reported on this data gathering experiment in [7].

We configured the WiFi sensors to capture all probe request frames. Once a frame was captured, the sensor created a detection with the tuple <*t*, *d*, *s*> where *t* is time, *d* device id (salted hash of the MAC address of the detected device), and *s* is the id of the sensor. We also recorded *n*, the sequence number of the probe request. We can use *n* to determine when two sensors receive the same frame.

### 5.2. Comparison

During the data gathering for the Assen festival, we conducted a small ground truth gathering, for a small number of devices. To achieve this, we walked around the festival, carrying with us nine WiFi devices (smartphones and tablets). We logged our locations at regular intervals and had a few of the devices gather GPS data. We verified the GPS locations with our notes and used the resulting dataset as the ground truth. For this dataset, we manually labeled the periods in which we moved and those in which we were not moving.

To compare the performance of the algorithms, we ran them on this limited dataset of nine devices and calculated the accuracy as compared to our list of stop and movement periods. We note that some inaccuracies may appear because of the manual labeling of the ground truth stop and movements set.

Each algorithm can be executed using multiple settings for their parameters.

- Cbsmot has maximumDistance and minimumTime, which combined control the maximum speed for which a period is to be considered a stop.
- Dbsmot has minimalDirectionChange, representing the tolerance boundary between a movement’s direction and the direction when the device is stopped, as well as a limit maxTol to how many consecutive points there need to be to form a cluster, meaning for a period to be considered stopped.
- Stay point detection has minimalDistance, representing the threshold distance from the pivot for a new detection to be considered movement.

After running the algorithms, we clean the resulting list of stops and movements using a maximalMovementDuration, representing the maximum amount of time for a period of two detections to be considered movement. The scope is to avoid labeling large gaps in detections as movement periods when there is not enough data to make any labeling. We also filter using a minimalStopDuration, which merges movements that have a gap between them smaller than this limit. The value is used to hide small stops, which we consider irrelevant, such as those at traffic lights. These short stop periods will clutter the dataset.

We executed the algorithms using multiple settings for each of these parameters, as can be seen in Table 1, and extracted lists of stop and move periods. We compared the lists with the ground truth to calculate the accuracy. Because we have a binary classification problem, only stops and movements, we could mark movement as positive and stops as negative. We then calculate the accuracy of identifying periods by comparing each second in the dataset. Detections can have large gaps, making it difficult to compare two different traces by comparing the labels of detections. We count a correctly-labeled move second as a true positive (TP) and an incorrectly labeled one as (FP). We do the same for stops and obtain true negatives and false negatives (TN/FN).

With each second labeled, we calculated the F1 score to determine the accuracy. To calculate the F1 score, we used Equation (1). We used F1 instead of accuracy because it considers both precision and recall, and we believe it better describes the differences between the algorithms.

(1) $$F1=\frac{2*TP}{2*TP+FP+FN}$$

**Figure 3:**
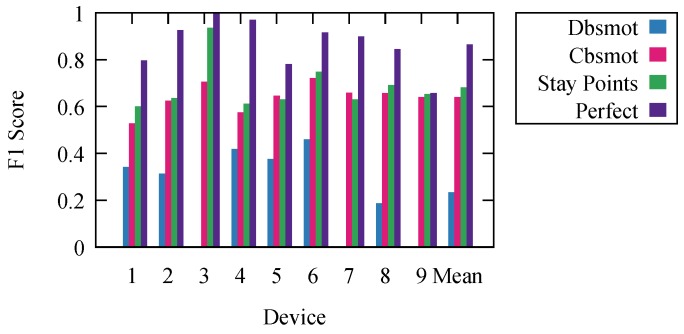
Comparing algorithms.

The resulting F1 scores are presented in Figure 3. They represent the results for each algorithm with the parameter settings that offer the highest mean F1 score.

To offer an even better understanding of the algorithms, we wanted to determine the maximal theoretical limit at which an ideal algorithm would perform. To do this, we calculated perfect results. We obtained them using the ground truth knowledge. We labeled each period based on the ground truth. However, because we calculated the F1 score for every second and consecutive periods could have any duration, due to the frequency of detections, the resulting stop/movement list, compared to the ground truth one, did not have an F1 score of one. It is impossible to develop an algorithm that would offer better results than the results labeled as perfect.

Stay point detection was the best performing algorithm. The worst performing was Dbsmot. Dbsmot did not perform well because of the small number of possible positions. This caused direction changes for WiFi-tracking datasets to be more often than they would be for GPS.

Even though Cbsmot and stay point detection offered similar results, we want to point out that the time complexity of stay point detection is better, and the runtime is significantly faster. Stay point detection is O(N), while Cbsmot is $O(N^{2})$; for our dataset, this means an execution time of under an hour as compared to several days.

## 6. Algorithm Robustness

It comes naturally that these algorithms are dependent on the walking speed and detection frequency. If the walking speed is very low, it might be difficult even for a human to determine if the trace represents a person moving or sitting still, especially with the low accuracy of WiFi positioning and sparsity of the data.

We want to explore the effects that variations in speed and detection frequency have on these three algorithms. Knowing accuracy estimates for different scenarios can lead to a more informed decision on the choice of algorithm or even the technology.

Setting up a real-life experiment in which human walking speed is precisely controlled for many people is not feasible. Instead, we built a synthetic dataset that aims to represent crowds for which we can control different features.

### 6.1. Generating a Synthetic Dataset

We created the synthetic dataset by simulating the movement of 100 individuals and recording detections at the sensors in their proximity. For a better comparison with the real-life results, we made use of the map of Assen city and assumed the sensors were placed in the same positions as they were during our festival experiment.

The simulated movement used a model similar to random walk. Everyone is randomly placed on the street map. We then select a random target position from the street map, to which the individual should walk, farther than 300 m from his/her current position (we chose the value to be 300 m by considering the size of the city and the ideal detection radius of WiFi). The individual then moves with a fixed speed towards the new position using the shortest path on the street map. Once the position is reached, a new position is chosen, and the process is repeated. To generate stops and movements, the individuals move continuously for one hour and then stay at the same location for the next hour. This is repeated for the 24 h of the simulation.

For our simulation, we assumed there was no interference for the WiFi signal. Detections were generated once per second for each individual at all sensors closer than 100 m from him/her.

To control the speed of movement, we split the individuals into groups of 10 people each and set the same walking speed for the entire group. The speeds were chosen between 0.1 m/s and 2 m/s. In contrast, the normal movement speed of a person is around 1.4 m/s. We used groups of 10 people to mitigate any issue caused by randomness in the movement.

In real life, the number of detections can be affected by the frame-transmission frequency, as well as frame loss caused by interference or environmental noise. To obtain a different number of detections, we sampled the resulting dataset. We kept between 0.01% and 100% of detections. The entire dataset had, on average, 2.5 detections per second for each device because most areas were covered by multiple sensors. When we sampled at 0.01%, we were left, on average, with two detections for every three minutes.

### 6.2. Results with Synthetic Data

We ran each of the algorithms on the generated datasets. We compared the results with the known stop and move periods and calculated the accuracy in determining the state for each second of the 24-h period.

We calculated the average accuracy for each speed setting and sampling rate. This means the values were representative of 10 synthetic traces each and accounted for randomness in the movement.

Because of the placement of sensors, it was possible for an individual to be outside of the detection area. The effect was even stronger if the individual stopped in one such location, creating movement periods of one hour that were labeled as stops in the ground truth list. This was one of the reasons why we did not expect perfect accuracy.

As presented in the previous sections, the algorithms can be executed using different values for their parameters. To have a fair comparison between the algorithms, we executed them with multiple parameter settings and present only the results for the set of values that resulted in the highest average accuracy.

**Figure 4:**
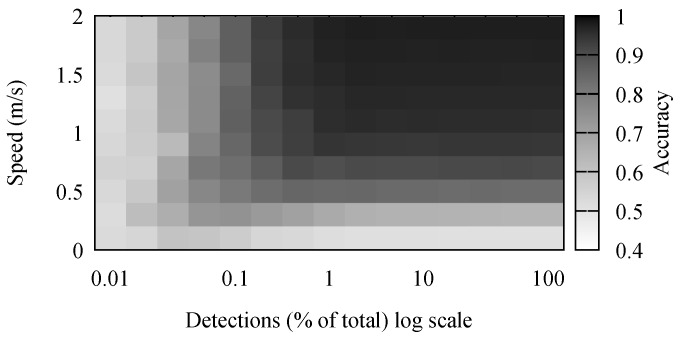
Effects of speed and detection frequency on the accuracy of stay point detection.

The accuracy results for stay point detection are presented in Figure 4. It comes naturally that the highest accuracy was achieved when the speed of the individual was high and there were many detections. It is simpler to differentiate between movement and stop periods when the movement has a high speed. Because of the low positioning accuracy, movements with a small speed appeared to the algorithm as a set of small stops combined with movements. Some of these were mitigated by the removal of short stop periods.

The number of detections had a noticeable, but small effect on the accuracy of the algorithm. However, in the simulation, detections were generated every second. Performing outdoor, large crowd, experiments, we observed this to be far from reality. In our outdoor experiments, detection frequencies were in the order of minutes, and there were few simultaneous detections. Therefore, the real-life traces were comparable to the simulated scenarios where between 0.1% and 1% of detections was available.

**Figure 5:**
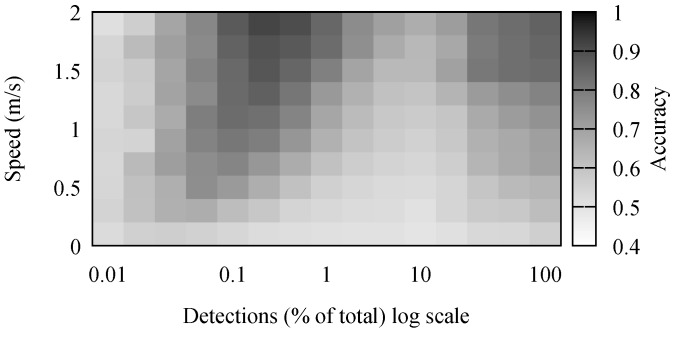
Effects of speed and detection frequency on the accuracy of Cbsmot.

An interesting result is offered by the Cbsmot algorithm. As we can see in Figure 5, there were two separate areas where the algorithm performed well. The algorithm found large clusters, meaning large stop periods. When the number of detections was low, more, smaller clusters were formed because consecutive detections were at sensors that were far apart. This improved the accuracy of the algorithm when detecting movements. In contrast, when there were many detections, the accuracy was high since many of them were simultaneous, forcing the separation of clusters, in turn separating the dataset into multiple periods of small movements.

## 7. Improvements on the Distance Function

We chose stay point detection as the algorithm to improve. It is the most accurate and simple, and we could easily modify it by replacing the distance function. The algorithm assumes the detection area for each sensor is a perfect disc. We know this is far from true, as buildings and other elements affect the range at which a device can be detected. Because of this, we wanted to replace the distance function with one based on the data.

The geographical distance was not ideal because individuals move on a limited network of streets and buildings modify the shape of the detection area.

We know that there was a limited set of sensors, and because each sensor represents a position, there is a limited set of possible positions in a WiFi tracking dataset. Because the set of sensors was limited and relatively small, we could calculate distances between all sensor pairs.

### 7.1. Definitions

We use the following notations:

*S*: the set of sensors $\left\{s_{1},\ldots ,s_{N}\right\}$ where one sensor is $s_{p}$ and a subset of sensors is $s_{P}$.

*D*: the set of devices $\left\{d_{1},\ldots ,d_{M}\right\}$.

*t*: time, measured in seconds.

Λ: the set of detections $\left\{\lambda_{1},\ldots ,\lambda_{R}\right\}$ where a detection is a tuple 〈s,d,t,n〉 (where *n* is the sequence number of the frame).

Λ[s]: the set of detections at sensor *s*. Analogous notations are Λ[d], Λ[t], Λ[n]. Similarly, Λ[d][s] represents the set of detections of device *d* at sensor *s*.

$\bar{\Lambda }=\left(\lambda_{1},\ldots ,\lambda_{R}\right)$: a sequence of detections so that they are ordered by device, time, sequence number, and finally, sensor.

$\bar{\Lambda [s]}=\left(\lambda_{0},\ldots ,\lambda_{P}\right)$: a sequence of consecutive detections at sensor s. It is a subset of $\bar{\Lambda }$ in which $\forall i;\lambda_{i}\left[s\right]=s$. We use an analogous notation for $\bar{\Lambda [d]}$.

### 7.2. Sensor Neighborhood Graphs

A graph with each sensor as a vertex and a weighted edge between each pair of vertices is a sensor distance graph (SDG). We denote such a graph SDG=(S,E). The weight of the edge represents the distance or closeness between the sensors at the vertices where it connects.

We set a threshold ϵ, and we defined the sensor neighborhood graph (SNG) as the subgraph of SDG, where all edges higher or lower than ϵ (depending on the distance or closeness function) are removed. The SNG can be used instead of a distance function for the stay point detection algorithm. If a device moved from one sensor to another and the two were not connected by an edge, we concluded the device must have moved. We used the following distance and closeness functions:

Geographical distance (DIS):

This is the classical distance function. We can measure the geographical distance between any two sensors and use it as the weight of the edge that connects the two. SNG contains the edges that have a small distance, so the ones that are smaller than ϵ.

(2) $$E=\{\left(s_{i},s_{j},w_{ij}\right)|\forall s_{i},s_{j}\in S;w_{ij}=\mathrm{GPSdistance}\left(s_{i},s_{j}\right)\}$$

Consecutive detections (CON):

We can assume that a person sitting still between, or moving between, two adjacent sensors generates consecutive detection at these two sensors. By using the tracking data, we counted how many times we had consecutive detections between any sensor pairs. The expectation here was that sensors that required a long path to reach would have fewer detections like these, compared to ones that had a shorter path. SNG kept all the edges that were close, meaning the ones that had values higher than a set ϵ threshold value. The next two closeness functions are based on this one, so the definition of SNG remains the same.

(3) $$\begin{array}{c} C\left(s_{i},s_{j}\right)=\left\{k\right|k\in \left[1,R\right];\exists \lambda_{k},\lambda_{k+1}\in \bar{\Lambda };\\ \lambda_{k}\left[s\right]=s_{i};\lambda_{k+1}\left[s\right]=s_{j};\lambda_{k}\left[d\right]=\lambda_{k+1}\left[d\right]\}\\ E=\{\left(s_{i},s_{j},w_{ij}\right)|\forall s_{i},s_{j}\in S;w_{ij}=|C\left(s_{i},s_{j}\right)|\} \end{array}$$

Simultaneous detections (SIM):

It is possible for two consecutive detections to have different or the same timestamps. For this method, we counted only detections that were also simultaneous. This implies that there was a point in time at which a device could be detected by the two sensors.

(4) $$\begin{array}{c} C1\left(s_{i},s_{j}\right)=\left\{k|k\in C\left(s_{i},s_{j}\right);\lambda_{k}\left[t\right]=\lambda_{k+1}\left[t\right]\right\}\\ E1=\{\left(s_{i},s_{j},w_{ij}\right)|\forall s_{i},s_{j}\in S;w_{ij}=|C1\left(s_{i},s_{j}\right)|\} \end{array}$$

Simultaneous detections validated with frame sequence number (SEQ):

It is possible for two detections to appear to be simultaneous because timestamps in our dataset had a precision of at most one second. To better confirm that two detections at different sensors represented the same frame, we can make use of the sequence number *n*.

(5) $$\begin{array}{c} C2\left(s_{i},s_{j}\right)=\left\{k|k\in C1\left(s_{i},s_{j}\right);\lambda_{k}\left[n\right]=\lambda_{k+1}\left[n\right]\right\}\\ E2=\{\left(s_{i},s_{j},w_{ij}\right)|\forall s_{i},s_{j}\in S;w_{ij}=|C2\left(s_{i},s_{j}\right)|\} \end{array}$$

Based on the definitions, we know C2⊆C1⊆C. This translates into weights from E2 being smaller than those in E1 and even smaller than the ones in *E*. In other words, the SNGs for the three graphs would have fewer edges for equivalent thresholds. An SNG with few edges can identify more refined movements. However, if there are not enough edges, any detection at a nearby sensor would force a label of movement.

We modified the stay point detection algorithm by replacing the distance function with an SNG. Movement only exists between two sensors not connected by an edge.

## 8. Improvement Experiments

Building distance/closeness graphs offers a few advantages to the algorithm: The distance between two points does not need to be calculated every time, as it is pre-calculated when building the graphs. This means the algorithm will run faster. The closeness graphs do not require information about the position of sensors as the closeness function is extracted from the dataset itself. More importantly, because the closeness measure is extracted from the dataset, it is more representative than any other distance function.

The graphs are different, and we need to understand the particularities of each one to understand how they will affect the stay point detection algorithm. We built multiple SNGs for each SDG and compared them. We chose pairs of SNGs (one from each SDG) by controlling the threshold ϵ so that we removed one edge at a time. This means that at first, we compared the SDGs and the full graphs, and at the end, we were left with graphs without any edges.

The full graphs, the SDGs, cannot be used to detect any movement. In contrast, for graphs with no edges, any detections at two sensors is considered a movement. When using a graph without any edges, for a device to be considered static, it would have to be detected by only one sensor.

To compare a pair of graphs, we count the number of edges they have in common. What this technique shows us is how much the order of edges arranged by weight changes from one SDG to the other. It also shows us where the order starts to differ.

**Figure 6:**
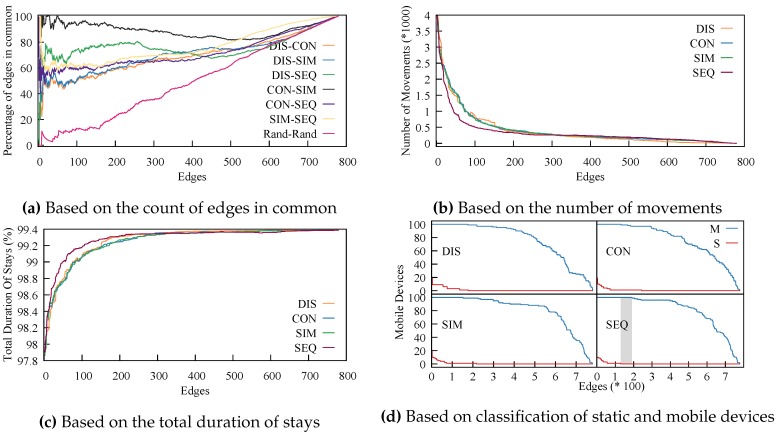
Comparing distance functions and graphs.

For each SDG, we sorted the edges by weight. Edges were sorted by increasing order for the distance graph and decreasing for the closeness graphs. We removed one edge at a time from the graphs and compared each pair of graphs. The results are plotted in Figure 6a. With full graphs, all edges are in common, regardless of what pair we chose to compare. This is why with an increased number of edges, the fraction of edges in common went to 100%. To offer a complete picture, we added a graph generated randomly. Rand-Rand went almost linearly from 0%–100%. All other pairs of graphs were above the Rand-Rand comparison, meaning there was a strong connection between all four graphs. This shows that although the closeness graphs did not rely on geographical distance, they were similar to the graph that represented geographical distance, and of course with each other; meaning, the connections formed because of geographical distance were still represented in the closeness methods.

The SDGs based on consecutive and simultaneous detections (CON-SIM) were the closest ones. Simultaneous detections represented a third of the consecutive ones. However, being close to each other was not an advantage; it just showed that it was not very relevant which of the two was used. An interesting point is that the distance graph was closest to the sequence numbers one (DIS-SEQ). This means that the SDG based on sequence numbers, constructed using only the dataset, was representative of the real world and the position of the sensors.

Next, we show that the SDG based on SEQ fits various scenarios and could prove to be even more useful than the SDG based on the geographical distance. To do this, we randomly selected 100 devices and ran the algorithm by removing one edge at time from the SDGs; of course, with the edges sorted by weight. In Figure 6b, we plot the number of movements, and in Figure 6c, we plot the total duration of movements. In both, SEQ stood out, although the difference was small, meaning the algorithm had similar performance regardless of the distance metric being used.

To better evaluate the effects of the different closeness functions on the algorithm, we compared them based on accuracy. To do this, we manually selected two groups of 100 devices each, one representing devices that we had a high confidence were static and yet were detected by multiple sensors. For the other, we selected devices we knew were mobile because they were detected by multiple distanced sensors. We selected these devices by visualizing the path created by tracing their detections. We denote the mobile group with M and the static one with S. The algorithm should detect at least one movement for each of the devices in group M and no movements for the devices in group S. Again, we controlled the number of edges in the SDGs. The results are presented in Figure 6d. The gray area is the only part where the algorithm achieves perfect accuracy. We had perfect accuracy only for the SEQ solution, when the threshold is set large enough to remove all false movement (intermittent detections at two or more adjacent sensors), yet small enough so as not to hide meaningful movement. Using this method, one can determine the correct threshold for a real-life application by utilizing test data from the real-life deployment or simulations. Using the other SDGs, the algorithm only gets close to the desired result, although the difference is minimal.

**Figure 7:**
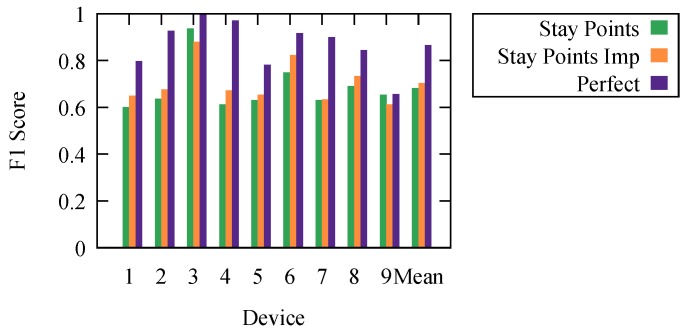
Comparing algorithms.

To confirm our results, we measured the effectiveness in determining the stops and movements for our nine devices, for which we had the ground truth. We executed the stay point detection algorithm using the SEQ graph and kept the results that offered the best mean F1 score. We present the results in Figure 7. Here, stay points and perfect are the ones from Section 5.

## 9. Conclusions

If we can understand crowd movements, we can open the way to multiple applications and improvements to various problems such as urban and facility planning. It is important to develop algorithms that facilitate understanding refined movement of large crowds of people. Just gathering data is not enough. We need to use algorithms to process them, and any step that can simplify the datasets can bring significant improvements because this allows us to tackle more complex questions.

We showed how algorithms previously designed for separating stops from movements in GPS tracking datasets performed once applied on WiFi-tracking datasets. We showed that at least one algorithm had an adequate performance, even though WiFi tracking data are less accurate and sparser than GPS tracking data. We then showed how to modify the best performing algorithm to further improve its results. The modifications increased the accuracy in partitioning, but also managed to replace the distance function. This means there was no longer a need for the location of devices to be known, making it simpler to deploy WiFi tracking platforms over already existing WiFi networks.

## Figures and Tables

**Table 1:** Parameter values.

Parameter Name	Values	Measuring Unit
maximumDistance	50, 60, ..., 490, 500	meters
minimumTime	60, 120, 300, 600	seconds
minimalDirectionChange	15, 30, 45, 60, 75, 90, 120, 150	degrees
maxTol	1, 2, 4, 6, 10, 20	
minimalDistance	50, 60, ..., 490, 500	meters
maximalMovementDuration	1800, 2700, 3600	seconds
minimalStopDuration	0, 20, ..., 260, 280, 300, 600, ..., 3300, 3600	seconds

## Abbreviations

The following abbreviations are used in this manuscript:

SDG	Sensor distance graph
SNG	Sensor neighborhood graph
DIS	Geographical distance
CON	Distance based on consecutive detections
SIM	Distance based on simultaneous detections
SEQ	Distance based on simultaneous detections with the same sequence number number
